# Trunk Lean Is Associated with the Magnitude of Forward Head Posture in Adolescents: A Cross-Sectional, Three-Dimensional Postural Analysis

**DOI:** 10.3390/jcm15197621

**Published:** 2026-10-01

**Authors:** Jung-Hoon Ahn, Yu-Sik Choi, Chang-Yoon Baek

**Affiliations:** 1 Department of Sports Rehabilitation, College of Health and Medical Science, Cheongju University, Cheongju-si 28503, Republic of Korea; 2 Department of Physical Therapy, College of AI-Bio Convergence, Yong In University, Yongin-si 17092, Republic of Korea; 3 Rehabilitation Center, National Health Insurance Ilsan Hospital, Goyang-si 10444, Republic of Korea; 4 Graduate School of Sports Medicine, CHA University, Pocheon-si 11160, Republic of Korea

**Keywords:** forward head posture (FHP), postural alignment, three-dimensional analysis, adolescents

## Abstract

**Background:** Forward head posture (FHP) is prevalent among adolescents and may occur as part of a broader pattern of three-dimensional (3D) postural alterations. Previous studies have primarily focused on isolated sagittal-plane measurements; therefore, the specific 3D postural characteristics associated with FHP remain unclear. This study investigated the 3D postural characteristics associated with FHP magnitude in adolescents. **Methods:** In this cross-sectional study, 256 adolescents were categorized into sample-based FHP tertiles (lower, middle, and upper) based on forward head displacement. Differences in postural variables among the tertiles were evaluated using age-adjusted multivariate analysis of covariance. Pearson correlation analyses with false discovery rate correction were used to examine associations among postural variables. Variables significantly associated with FHP were further examined using univariate and multivariable linear regression models. **Results:** After adjustment for age, shoulder protraction differed only between the lower and upper FHP tertiles, whereas trunk lean differed significantly across all tertiles. FHP was significantly correlated with trunk lean (r = 0.409) but not with shoulder protraction. In the multivariable regression analysis, trunk lean remained associated with FHP after adjustment for the other included variables (B = 0.408, BCa 95% CI: 0.279–0.526, *p* = 0.001), whereas the other postural variables did not. **Conclusions:** Among the evaluated 3D postural variables, trunk lean showed a consistent association with FHP magnitude in adolescents. However, this relationship should be interpreted cautiously because FHP and trunk lean share the shoulder landmark in their measurement definitions. These findings support consideration of sagittal trunk alignment when characterizing postural patterns associated with FHP in adolescents.

## 1. Introduction

Forward head posture (FHP) is among the most prevalent postural deviations observed in adolescents and is increasingly linked to prolonged digital device use and sedentary behaviors [1,2]. FHP is associated with a range of musculoskeletal complications, including neck pain, altered cervical biomechanics, and impaired sensorimotor control [2,3,4]. Adolescence is a critical period for musculoskeletal development; therefore, early identification of postural deviations such as FHP is essential to prevent long-term dysfunction.

FHP is traditionally evaluated using sagittal-plane parameters, such as the craniovertebral angle and forward head displacement, which primarily reflect anterior translation of the head relative to the trunk [3,4]. Although these measurements are simple and widely used, they assume that postural deviations occur predominantly within a single plane. However, human posture is a complex, three-dimensional (3D) construct involving coordinated interactions among multiple body segments rather than isolated joint alignments [5,6].

Evidence indicates that FHP is not an isolated cervical deviation but part of a broader postural pattern involving thoracic kyphosis, scapular positioning, and trunk alignment [7,8,9]. However, previous studies have primarily focused on individual segments or two-dimensional assessments, limiting our understanding of the integrated 3D nature of postural control. In particular, previous theoretical frameworks have proposed that trunk orientation is closely related to head alignment, on the premise that changes in trunk orientation may shift the center of mass and be accompanied by adjustments in head position to maintain visual and vestibular stability [10,11]. Whether such a relationship is observable in the habitual standing posture of adolescents, however, has not been fully examined. Since body segments are mechanically linked through the kinetic chain, frontal- and transverse-plane malalignments (e.g., shoulder-height asymmetry or trunk rotation) may also be related to sagittal head position. Therefore, a multi-plane assessment is required to determine which segments are most relevant to FHP magnitude.

Moreover, objective assessments of the 3D postural characteristics associated with FHP remain limited, particularly in adolescents. Many studies rely on visual assessments or isolated biomechanical variables, failing to adequately capture the complex interrelationships among body segments [12,13]. Identifying the postural components independently associated with FHP is clinically important, as it may facilitate the development of more targeted and effective physical therapy interventions.

Therefore, this study aimed to investigate the 3D postural characteristics associated with the magnitude of FHP in adolescents using objective measurement techniques. Rather than prespecifying a dominant postural contributor, we comprehensively examined multiple segments to identify which components are independently associated with FHP [14,15,16].

Given the previously proposed kinematic chain relationship between trunk and head alignment [5,10,11], we hypothesized that among the evaluated body-segment parameters, sagittal trunk alignment (trunk lean) would be most strongly associated with FHP magnitude, whereas frontal- and transverse-plane parameters would demonstrate weak or no independent associations after accounting for trunk alignment.

## 2. Materials and Methods

### 2.1. Participants

In this cross-sectional study, 256 adolescents were recruited from local schools through convenience sampling. Adolescents aged 14–17 years who were able to independently maintain an upright standing posture were eligible for inclusion. Exclusion criteria included (1) a history of neurological or musculoskeletal disorders affecting posture, (2) previous spinal or orthopedic surgery, and (3) current musculoskeletal pain or recent injury, as evaluated via a standardized self-report questionnaire completed with a legal guardian. All participants and their legal guardians provided written informed consent before participation. The study protocol was approved by the Institutional Review Board of the National Health Insurance Ilsan Hospital, Republic of Korea (approval number: NHIMC 2026-03-014), and conducted in accordance with the Declaration of Helsinki.

### 2.2. Study Design

FHP was quantified in 256 adolescents using a 3D postural assessment system. For descriptive group comparisons, participants were classified into three sample-based FHP tertiles according to the distribution of forward head displacement in the present sample: lower tertile (*n* = 84), middle tertile (*n* = 88), and upper tertile (*n* = 84). These tertile cutoffs were derived solely from the sample distribution to produce approximately equal group sizes and do not represent established clinical thresholds or severity categories. The tertile-based comparisons were considered secondary descriptive analyses, whereas correlation and regression analyses using FHP as a continuous variable constituted the primary analyses of association. Relationships between postural variables and FHP magnitude were evaluated using correlation and multivariable regression analyses to identify variables associated with FHP magnitude after adjustment for other postural variables.

### 2.3. Three-Dimensional Postural Assessment

**Figure 1:**
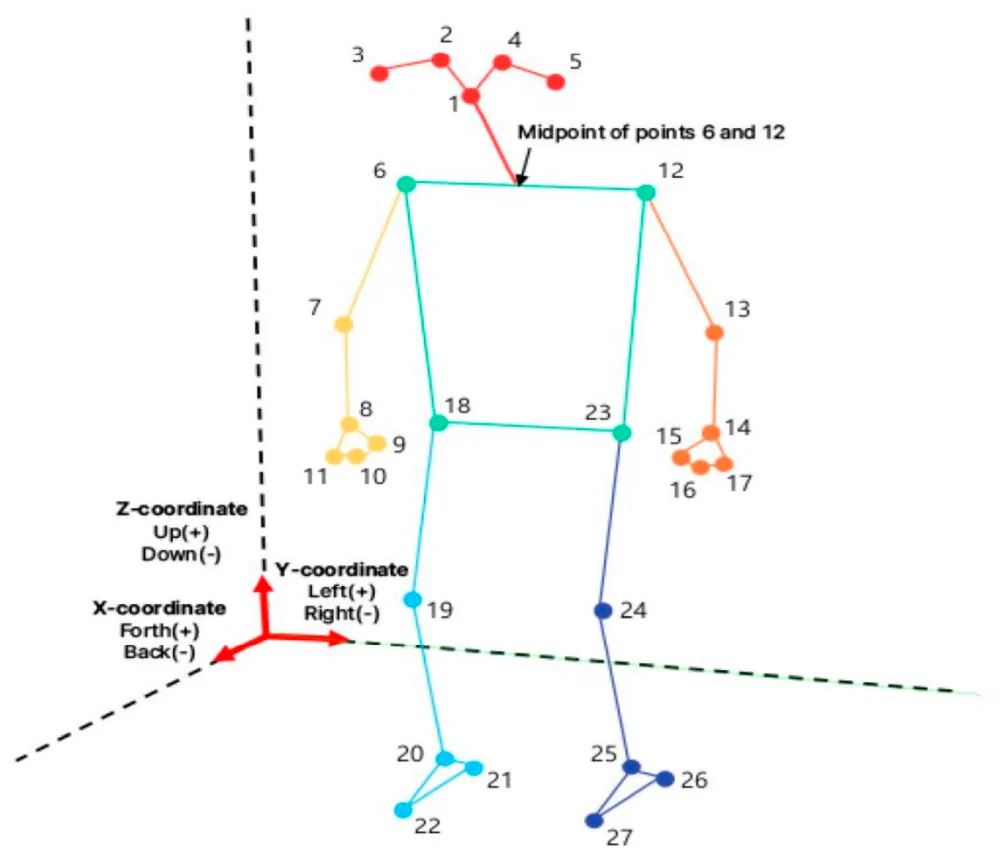
Anatomical landmarks and the coordinate system used for three-dimensional postural analysis. A total of 27 anatomical landmarks were automatically detected using the markerless multi-view RGB system. The X-, Y-, and Z-axes indicate the anterior–posterior, left–right, and vertical directions, respectively. The midpoint between the right and left shoulder landmarks is shown as a reference point.

**Figure 2:**
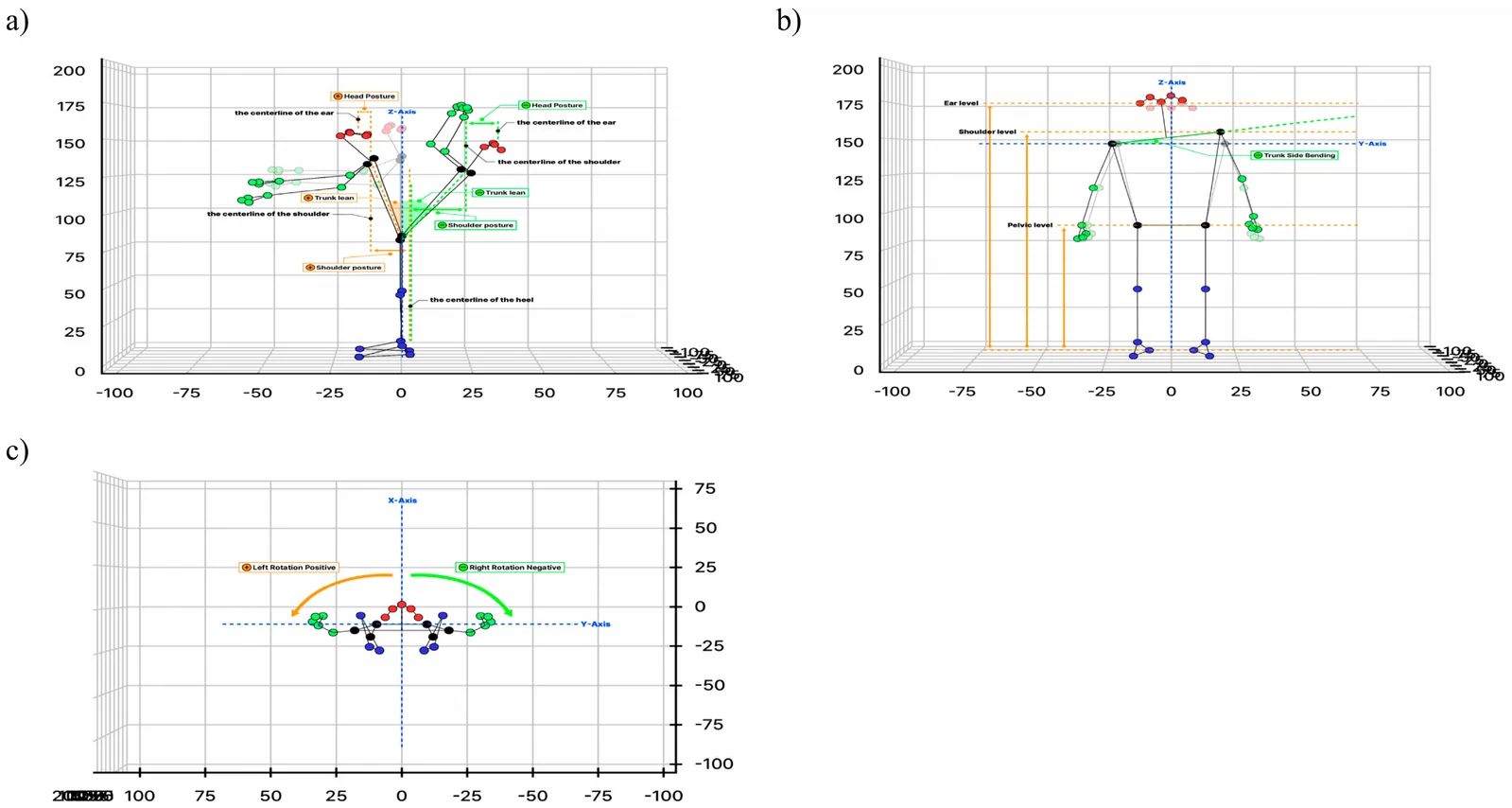
Three-dimensional reconstruction of postural alignment and sign conventions based on a markerless motion capture system. (**a**) Sagittal-plane variables: positive values indicate anterior displacement or anterior inclination, whereas negative values indicate posterior displacement or posterior inclination. (**b**) Frontal-plane variables: positive values indicate leftward deviation, and negative values indicate rightward deviation. (**c**) Transverse-plane variables: positive values indicate leftward rotation, and negative values indicate rightward rotation.

Three-dimensional postural alignment was assessed using a markerless multi-view RGB system (4DEYE^®^, SYM Healthcare Inc., Seoul, Republic of Korea). The system consists of five synchronized RGB cameras and enables reconstruction of body alignment without physical markers. Images captured from multiple directions were processed using an image-analysis algorithm based on OpenPose and OpenCV. Anatomical keypoints were first detected as two-dimensional coordinates from each camera view, and the corresponding 3D landmark coordinates were reconstructed based on the relative positions and orientations of the cameras [6]. A total of 27 anatomical landmarks, including landmarks of the head, shoulders, trunk, pelvis, and lower extremities, were automatically detected, allowing quantitative postural analysis using a skeletal model (Figure 1). The reconstructed landmarks were then used to calculate postural variables according to predefined measurement planes and reference lines, as illustrated in Figure 2 and described below. This system has demonstrated acceptable intra-rater reliability (ICC3,1 = 0.773–0.974) and concurrent validity compared with the Vicon motion capture system (ICC3,k = 0.747–0.936) [17].

A comparable static standing assessment protocol using the same 4DEYE system has also been reported previously [6].

All parameters were measured under standardized laboratory conditions. Before each data-collection session, the 4DEYE system was calibrated according to the manufacturer’s procedures, and camera synchronization and spatial alignment were verified. Participants were assessed individually while standing on a platform (60 cm × 60 cm) positioned approximately 2 m from the front camera. They were instructed to maintain a relaxed, habitual standing posture with their feet shoulder-width apart and their arms resting naturally at their sides for 2–3 s. Each measurement was performed twice, with a 30-s rest interval between trials, and the mean value was used in the analysis. Because the OpenPose-based algorithm identifies keypoints from image appearance rather than from physical markers, clothing thickness and soft-tissue thickness over the ear, shoulder, and hip regions can displace the detected keypoint relative to the underlying bony landmark [18,19]. To reduce these sources of error, participants wore fitted clothing, standing position and camera calibration were standardized, and the mean of two trials was used to reduce trial-to-trial variability in keypoint detection. Participants were instructed not to intentionally correct their posture. The assessment area was enclosed with partitions, and external light sources were controlled to minimize measurement error.

### 2.4. Outcome Variables

Three-dimensional postural parameters derived from the assessment system were analyzed; these included FHP, shoulder protraction, trunk lean, eye level, shoulder level, pelvic level, trunk-side bending, and segmental rotation of the head, trunk, and pelvis. Sagittal-plane variables were calculated separately for the left and right sides using 3D landmarks projected onto the sagittal plane. In the sagittal plane, FHP was defined as the horizontal distance (cm) between the ear and shoulder landmarks during quiet standing. Shoulder protraction was operationally defined according to the predefined 4DEYE measurement protocol as the horizontal distance (cm) between the shoulder and heel landmarks in the sagittal plane. In this system, the heel serves as the distal reference landmark for quantifying the anterior–posterior position of the shoulder during quiet standing. Accordingly, this variable reflects anterior shoulder displacement within whole-body sagittal alignment rather than isolated scapular protraction [6]. Trunk lean was defined as the angle between the vertical reference line and the line connecting the shoulder and hip landmarks. Since the right- and left-side trunk lean values were identical in the present dataset, trunk lean was treated and presented as a single variable. Bilateral postural measures were analyzed separately because the 3D postural assessment system provided distinct right- and left-sided measurements. These side-specific values were retained rather than combined into averaged bilateral values to preserve potential postural asymmetry. Averaged or derived asymmetry measures were not prespecified outcomes of the present study. Positive values indicated anterior displacement for distance variables and anterior inclination for angular variables, whereas negative values indicated posterior displacement or posterior inclination. For trunk lean, values closer to zero indicate a smaller degree of posterior trunk inclination.

In the frontal plane, eye level, shoulder level, and pelvic level represented the vertical positions of the corresponding anatomical landmarks relative to the ground reference line. Trunk-side bending was quantified as the frontal-plane angle between the horizontal reference line and the line connecting the bilateral shoulder landmarks. According to the sign convention shown in Figure 2, negative values indicate rightward deviation, whereas positive values indicate leftward deviation. Segmental rotations of the head, trunk, and pelvis were analyzed in the transverse plane, with positive values indicating leftward rotation and negative values indicating rightward rotation.

### 2.5. Statistical Analysis

All statistical analyses were performed using IBM SPSS Statistics (version 18.0; IBM Corp., Armonk, NY, USA). Categorical baseline characteristics were compared using the chi-squared test, whereas continuous baseline characteristics were compared among the sample-based FHP tertiles using one-way analysis of variance (ANOVA). Three-dimensional postural variables, excluding FHP itself because it was used to define the tertiles, were compared among the sample-based FHP tertiles using multivariate analysis of covariance (MANCOVA), with age included as a covariate because it differed significantly among the tertiles and may influence postural alignment during adolescence. When the overall multivariate effect was significant, follow-up univariate ANCOVAs were performed for the individual postural variables, followed by Bonferroni-adjusted pairwise comparisons where appropriate. Assumptions for MANCOVA were assessed using Box’s M test for homogeneity of covariance matrices and Levene’s tests for equality of error variances. Because the homogeneity of covariance matrices assumption was violated, Pillai’s Trace was used as the primary multivariate statistic because of its greater robustness to covariance heterogeneity.

Pearson’s correlation analyses were performed to characterize the bivariate relationships between FHP and all 13 postural variables, as well as the interrelationships among these variables. To reduce the risk of type I error due to multiple comparisons, *p* values from the correlation analyses were adjusted using the Benjamini–Hochberg false discovery rate (FDR) procedure.

For the regression analyses, univariate linear regression analyses were performed separately for all 13 postural variables to examine their individual associations with FHP. Variables showing significant associations in the univariate analyses were considered candidate variables for the multivariable model to reduce model complexity and avoid overparameterization. Age was retained as a covariate because of its potential influence on postural alignment during adolescence. The final multivariable linear regression model included the selected candidate variables and age as a covariate. Regression assumptions were assessed by visual inspection of standardized residual plots and normal P–P plots for linearity, homoscedasticity, and normality of residuals. Independence of residuals was evaluated using the Durbin–Watson statistic, and influential observations were assessed using Cook’s distance. Because bilateral measures may represent closely related anatomical constructs, their inclusion in the same multivariable model was evaluated for multicollinearity. Multicollinearity was assessed using variance inflation factors (VIFs), with VIF values greater than 10 considered indicative of severe multicollinearity, consistent with a commonly used conventional threshold, while recognizing that lower cutoffs have also been proposed [20]. Since the Rt. and Lt. pelvic levels showed severe multicollinearity when entered simultaneously, only the Lt. pelvic level was retained in the final model, and the Rt. pelvic level was examined separately in a sensitivity model to assess the robustness of the results. Additional sensitivity analyses were performed with further adjustment for sex, height, and body mass index (BMI). To enhance the robustness of the final multivariable regression estimates, participant-level bootstrap resampling with 1000 samples was performed to obtain bias-corrected and accelerated (BCa) 95% confidence intervals and bootstrap *p* values.

Analyses using FHP as a continuous variable were considered the primary analyses of association, whereas the tertile-based group comparisons were considered secondary exploratory analyses. Multiplicity was addressed using the Benjamini–Hochberg FDR procedure for the correlation analyses and Bonferroni adjustment for pairwise comparisons following MANCOVA. The multivariable regression model was considered the primary adjusted analysis, whereas the univariate screening and sensitivity analyses were considered exploratory and were not subjected to additional multiplicity correction.

Based on a G*Power 3.1 estimation for multiple linear regression (fixed model, R^2^ deviation from zero), a minimum sample size of 131 participants was required, assuming 13 predictors, a medium effect size (f^2^ = 0.15), α = 0.05, and a statistical power of 0.80, according to Cohen’s recommendations for multiple regression analyses [21]. For the correlation analysis, the assumed effect size was informed by a previous posture-related study showing an association between thoracic kyphosis and FHP (Spearman’s ρ = −0.48) [9]. The sample-size calculation for multiple regression was intended to support that specific analysis and should not be interpreted as establishing adequate statistical power for all analyses performed in the study.

## 3. Results

### 3.1. Participant Characteristics

A total of 256 adolescents were included in the analysis and categorized into the lower (*n* = 84), middle (*n* = 88), and upper FHP tertiles (*n* = 84). Age differed significantly among the three tertiles (F = 4.061, *p* = 0.018), with the upper FHP tertile exhibiting a slightly higher mean age (Table 1). Sex distribution (χ^2^ = 0.200, *p* = 0.905), height (*p* = 0.320), weight (*p* = 0.112), and BMI (*p* = 0.164) did not differ significantly among the tertiles.

### 3.2. Group Differences in Postural Variables Across FHP Tertiles

After adjusting for age, the overall multivariate effect of FHP tertile on the postural variables was significant (Pillai’s Trace = 0.357, F(26, 482) = 4.031, *p* < 0.001, partial η^2^ = 0.179). Pillai’s Trace was used because Box’s M test indicated a violation of the homogeneity of covariance matrices assumption (*p* < 0.001). Levene’s tests indicated some violations of the homogeneity of error variance assumption. Given the nearly equal group sizes across the tertiles, the follow-up univariate ANCOVA results were retained and interpreted with caution. Subsequent univariate ANCOVAs showed significant differences among the tertiles in right shoulder protraction (F(2, 252) = 3.826, *p* = 0.023, partial η^2^ = 0.029), left shoulder protraction (F(2, 252) = 3.564, *p* = 0.030, partial η^2^ = 0.028), and trunk lean (F(2, 252) = 25.886, *p* < 0.001, partial η^2^ = 0.170) (Table 2). Bonferroni-adjusted pairwise comparisons revealed that the upper FHP tertile demonstrated greater right shoulder protraction than the lower FHP tertile (*p* < 0.05), whereas the middle FHP tertile did not differ significantly from either of the other two tertiles. For left shoulder protraction, a significant difference was observed only between the lower and upper FHP tertiles (*p* < 0.05). Trunk lean values were significantly lower in the lower FHP tertile than in the middle FHP tertile (*p* < 0.001) and the upper FHP tertile (*p* < 0.001) and significantly lower in the middle FHP tertile than in the upper FHP tertile (*p* < 0.01). Because negative trunk lean values indicate posterior trunk inclination, participants in the lower FHP tertile showed relatively greater posterior trunk inclination.

### 3.3. Pearson Correlations Among Head Posture, Shoulder Protraction, Trunk Lean, and Other Postural Variables

**Figure 3:**
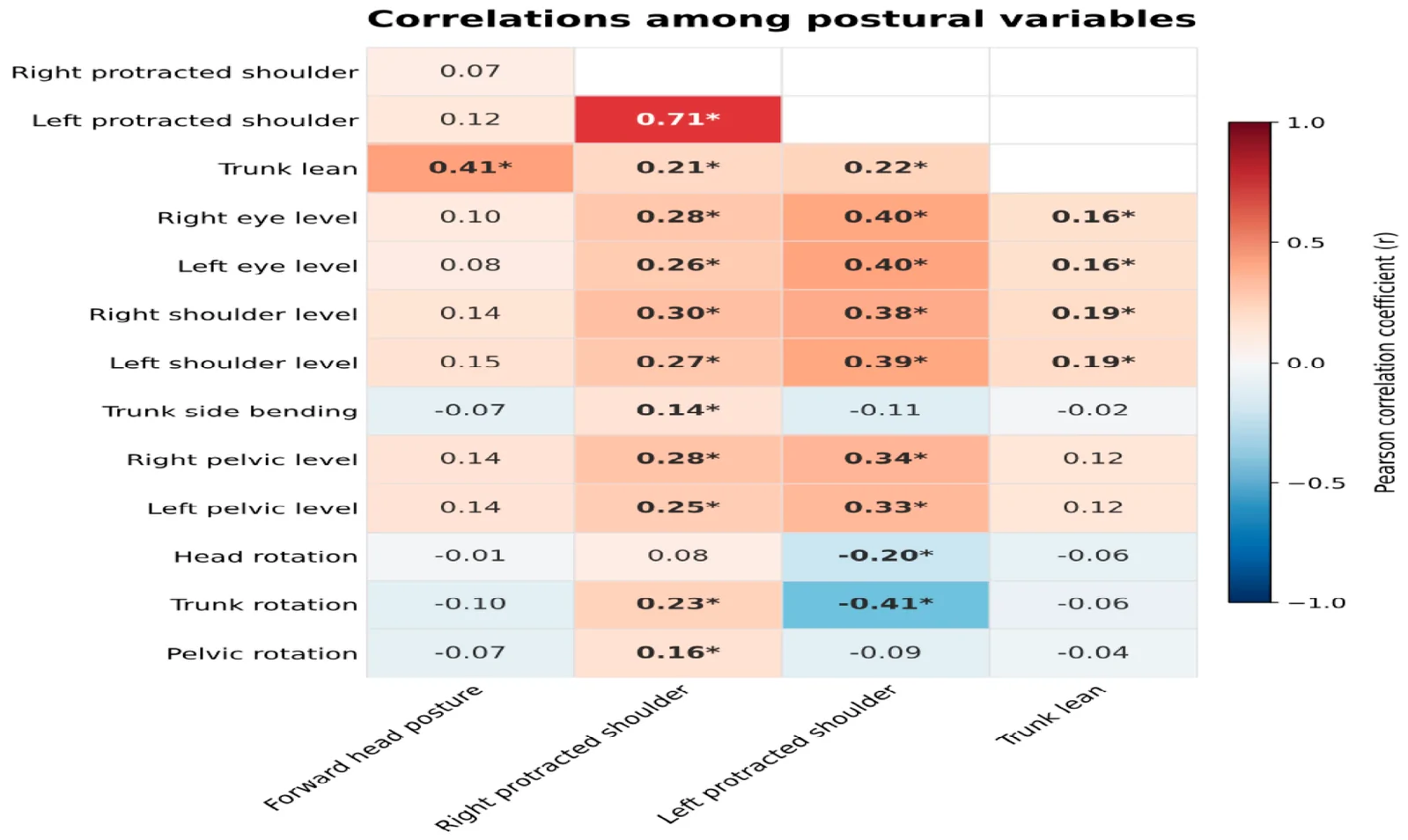
Pearson correlations among postural variables. Heatmap illustrating Pearson correlation coefficients between FHP, right and left shoulder protraction (Rt PS and Lt PS), trunk lean, and additional postural alignment variables. Red indicates positive correlations, whereas blue indicates negative correlations; color intensity reflects the strength of the association. Numerical values within each cell represent Pearson correlation coefficients (*r*). Asterisks (*) indicate correlations that remained statistically significant after Benjamini–Hochberg false discovery rate (FDR) correction (FDR-adjusted *p* < 0.05).

Pearson’s correlation analysis was performed (Figure 3). Statistical significance was determined using the Benjamini–Hochberg FDR procedure, with an FDR-adjusted *p* < 0.05 considered statistically significant. FHP was significantly correlated with trunk lean (r = 0.409) but not with shoulder protraction. Right shoulder protraction was significantly correlated with left shoulder protraction (r = 0.710), trunk lean (r = 0.208), right eye level (r = 0.277), left eye level (r = 0.259), right shoulder level (r = 0.301), left shoulder level (r = 0.266), right pelvic level (r = 0.283), left pelvic level (r = 0.254), trunk rotation (r = 0.230), and pelvic rotation (r = 0.156).

Left shoulder protraction was significantly correlated with trunk lean (r = 0.221), right eye level (r = 0.397), left eye level (r = 0.401), right shoulder level (r = 0.377), left shoulder level (r = 0.392), right pelvic level (r = 0.343), left pelvic level (r = 0.330), head rotation (r = −0.197), and trunk rotation (r = −0.407).

Trunk lean was also correlated with right eye level (r = 0.160), left eye level (r = 0.160), right shoulder level (r = 0.187), and left shoulder level (r = 0.188).

### 3.4. Linear Regression Analysis for FHP

Univariate linear regression analyses were conducted to identify variables associated with FHP (Table 3). Trunk lean was significantly associated with FHP (B = 0.416, 95% CI: 0.298–0.535, β = 0.401, *p* < 0.001). Left shoulder level (B = 0.038, 95% CI: 0.002–0.075, *p* = 0.041), right pelvic level (B = 0.054, 95% CI: 0.005–0.104, *p* = 0.031), and left pelvic level (B = 0.055, 95% CI: 0.005–0.104, *p* = 0.030) were also significantly associated with FHP in the univariate analyses.

The final multivariable model was significant (F(4, 251) = 13.734, *p* < 0.001), explaining 18.0% of the variance in FHP (R^2^ = 0.180, adjusted R^2^ = 0.166). Regression diagnostics indicated no major violations of linearity, normality, homoscedasticity, or independence of residuals. The Durbin–Watson statistic was 1.788, and the maximum Cook’s distance was 0.041, indicating no substantial autocorrelation or influential observations. After adjustment for age and the other included variables, trunk lean remained significantly associated with FHP (B = 0.408, BCa 95% CI: 0.279–0.526, β = 0.393, *p* = 0.001), whereas left shoulder and left pelvic levels were not significantly associated. Because negative trunk lean values indicate posterior inclination, the positive regression coefficient indicates that greater FHP was associated with a less posteriorly inclined, more anteriorly oriented trunk. In the sensitivity analysis additionally adjusted for sex, height, and BMI, the association between trunk lean and FHP remained significant (B = 0.412, BCa 95% CI: 0.284–0.534, *p* = 0.001), indicating that the observed association was maintained after accounting for sex- and body-size-related factors (Table 4).

## 4. Discussion

This study investigated the 3D postural characteristics associated with FHP in adolescents and identified trunk lean as the postural variable most consistently associated with FHP magnitude across the analytical approaches used. Trunk lean demonstrated a moderate association with FHP, exhibited graded differences across the FHP tertiles, and remained significantly associated with FHP after adjustment for age and the other included postural variables. Collectively, these cross-sectional findings indicate that FHP magnitude was statistically associated with the measured sagittal trunk orientation in this adolescent sample. In the follow-up ANCOVAs, although shoulder protraction differences were statistically significant, their effect sizes were small (partial η^2^ = 0.029 and 0.028), suggesting that their practical relevance may be limited. Trunk lean showed a larger effect (partial η^2^ = 0.170) and a moderate standardized association in the multivariable model (β = 0.393); however, the clinical significance of these findings remains uncertain and should be interpreted cautiously because of the shared shoulder landmark with FHP. Although the cross-sectional design cannot establish directionality, a potential biomechanical explanation is that sagittal trunk inclination and head position may be coordinated as part of whole-body postural alignment. Previous biomechanical frameworks have described interactions among adjacent body segments during postural control [5,14]. However, the present findings do not establish that trunk inclination causes or mechanically determines forward head displacement. Moreover, because all variables were obtained from a single static assessment, the direction of any relationship between trunk and head position and whether either represents a compensation for the other cannot be inferred from these data.

Significant interrelationships were observed among several postural variables, suggesting coordinated relationships among multiple body segments. However, these associations did not uniformly correspond to FHP magnitude. In the present dataset, a consistent association with FHP was observed for sagittal trunk lean. Three-dimensional assessment of adolescent posture has previously been undertaken using rasterstereography and photogrammetry-based approaches [22,23], and age-related changes in sagittal posture parameters have been described in children and adolescents [24]. However, relatively few studies have simultaneously quantified sagittal-, frontal-, and transverse-plane segmental alignment together with FHP in a large adolescent sample using a markerless multi-view RGB system; the present study adds to this literature rather than establishing a new paradigm. The inclusion of multiple planes allowed the relationship between FHP and trunk lean to be examined within a broader 3D postural context.

The current findings are generally consistent with previous studies reporting associations between FHP and thoracic kyphosis or trunk flexion [7,9]. Although trunk lean, which reflects sagittal trunk orientation, differs geometrically from traditional measures of thoracic kyphosis, these findings collectively suggest that sagittal trunk characteristics may be related to head position. Importantly, trunk lean remained significantly associated with FHP after adjustment for the other included variables. However, because FHP and trunk lean share the shoulder landmark in their measurement definitions, the magnitude of this association may partly reflect shared measurement geometry and should therefore be interpreted cautiously.

The measurement coupling between FHP and trunk lean warrants explicit consideration. In the present system, FHP corresponds to the anterior–posterior offset of the ear landmark relative to the shoulder landmark, whereas trunk lean is the inclination of the line connecting the shoulder and hip landmarks from the vertical reference. Because the anterior–posterior coordinate of the same shoulder landmark enters both definitions, the two variables are not mathematically independent. Under the sign conventions used, an isolated anterior displacement of the shoulder landmark, with the ear and hip landmarks fixed, would decrease FHP while increasing trunk lean; random localization error at the shoulder alone would therefore be expected to induce a negative covariance and to attenuate rather than inflate a positive association. Conversely, coordinated anterior displacement of the shoulder and head relative to the hip would increase both variables and produce a positive association that is consistent with true sagittal postural coupling. Because the present dataset does not include a trunk measure that is independent of the shoulder landmark, the proportion of the observed association attributable to measurement geometry rather than postural coupling cannot be quantified. Future studies could address this by deriving trunk orientation from landmarks that do not enter the FHP definition (e.g., a hip–C7 or hip–sternal reference) and by simulation-based sensitivity analyses that propagate landmark uncertainty through the variable definitions.

The regression analysis provided additional evidence of an adjusted association between trunk lean and FHP. After adjustment for age and the other included postural variables, trunk lean remained statistically significant, whereas left shoulder and left pelvic levels were no longer significant. These findings indicate that the associations of these variables with FHP were attenuated after multivariable adjustment; however, the present analysis cannot determine whether this attenuation resulted from their relationships with trunk posture or from other sources of shared variance. More broadly, similar interrelationships among postural variables have been discussed in the context of proximal-to-distal kinetic chain concepts in previous studies [25,26,27].

From a clinical perspective, the present findings suggest that sagittal trunk alignment may be considered when characterizing postural patterns associated with FHP in adolescents. Objective 3D postural assessment may provide additional information regarding broader postural characteristics beyond localized head and shoulder measurements. However, because of the cross-sectional design and the shared measurement geometry between FHP and trunk lean, these findings should not be interpreted as evidence that modifying trunk alignment would reduce FHP. Longitudinal studies using anatomically independent measures are needed to clarify the clinical relevance of the observed relationship. As markerless motion-capture and other digital assessment technologies become increasingly integrated into musculoskeletal practice, future clinical implementation will also require attention to the secure management, interoperability, and traceability of longitudinal digital health data. Emerging technologies such as blockchain have been proposed in orthopedics as possible tools to improve data integrity and information sharing within increasingly digital clinical pathways [28]. Such considerations will be relevant if objective postural assessment of adolescents is to be repeated over time and shared across educational and clinical settings.

This study has several strengths. A digital 3D postural assessment system enabled objective and comprehensive quantification of postural variables across multiple anatomical planes. In addition, the use of complementary analytical approaches, including tertile-based comparisons, correlation analyses, and multivariable regression, allowed the relationship between FHP and postural variables to be examined from several statistical perspectives.

However, this study also has several limitations. First, its cross-sectional design precludes causal inference; the observed associations cannot establish whether trunk alignment influences head position or vice versa. Second, the shared shoulder landmark between FHP and trunk lean represents a potential measurement dependency. FHP was calculated as the horizontal distance between the ear and shoulder landmarks, whereas trunk lean was calculated as the angle of the shoulder–hip line relative to the vertical reference line. Because this landmark contributes to both measurements, the observed association may partly reflect shared measurement geometry rather than solely sagittal-plane postural coupling. An alternative trunk measure based on non-overlapping anatomical landmarks was not available in the present dataset; therefore, this relationship should be interpreted cautiously, and future studies should use independent anatomical reference points to verify the observed association. Third, several potentially important confounders were not measured or controlled. Daily screen time and digital device exposure, physical activity level and sporting participation, habitual sitting posture, and history of neck pain were not recorded, although computer and mobile device use has been associated with habitual spinal posture and neck flexion in adolescents [29,30]. Because the timing of the assessment relative to preceding device use or sedentary activity was not standardized, immediate postural fatigue or short-term postural adaptation could not be excluded as an influence on the static standing measurements. In addition, pubertal or maturational status was not assessed. Because sagittal posture parameters change with growth and maturation [24], the narrow chronological age range of the sample may not fully capture this variation. These unmeasured factors could partially explain or modify the observed relationship between FHP and trunk alignment. Fourth, participants were recruited by convenience sampling from local schools within a single geographic region of Korea, and the age range was relatively narrow (14–17 years). The findings may therefore not generalize to younger or older adolescents, to adolescents outside the school setting, or to populations with different anthropometric characteristics, cultural postural habits, or patterns of digital device use. The sample-based tertile cutoffs, in particular, should not be applied to other populations. Fifth, FHP and shoulder protraction are distance-based measures that may be influenced by body size. Although height, weight, and BMI did not differ significantly among the FHP tertiles, the use of absolute distance measures should be considered when interpreting these findings. Furthermore, although fitted clothing and standardized conditions were used, residual soft-tissue and clothing artifacts in automated landmark detection cannot be excluded. However, BMI did not differ across the FHP tertiles, and the association between trunk lean and FHP was essentially unchanged after additional adjustment for sex, height, and BMI (B = 0.412 vs. 0.408; Table 4), suggesting that body composition-related landmark bias is unlikely to fully account for the main finding. Sixth, the postural assessment was limited to static standing and may not reflect dynamic functional alignment. Seventh, the multivariable regression model used a data-driven variable-selection strategy based on univariate statistical significance, which may have introduced model-selection bias and excluded variables that could become relevant after multivariable adjustment. Finally, although the tertile-based classification of FHP produced near-equal sample sizes, the cutoffs were sample-specific and do not represent established clinical thresholds or severity categories. Given the number of statistical comparisons and the exploratory nature of several secondary analyses, these findings should be interpreted cautiously and confirmed in independent samples.

## 5. Conclusions

In this cross-sectional sample of adolescents, the magnitude of forward head posture was statistically associated with the measured sagittal trunk lean parameter, and this association persisted after adjustment for age and the other included postural variables and was consistent across the complementary analytical approaches used. However, because FHP and trunk lean share the shoulder landmark in their measurement definitions, and because only static standing posture was assessed, the observed relationship may partly reflect shared measurement geometry and cannot be interpreted as evidence of an independent biomechanical or causal relationship. Accordingly, the present findings do not establish that trunk alignment is a determinant of FHP, nor do they indicate that trunk-focused interventions would modify FHP. Sagittal trunk orientation may nonetheless be a relevant parameter to include when characterizing postural patterns associated with FHP in adolescents. Longitudinal and interventional studies using non-overlapping anatomical reference points are needed to clarify the nature and clinical relevance of this association.

## Figures and Tables

**Table 1:** Characteristics of participants.

Characteristic	Total (*n* = 256)	Lower FHP Tertile (*n* = 84)	Middle FHP Tertile (*n* = 88)	Upper FHP Tertile (*n* = 84)	F/χ^2^	*p*-Value
Age, years	16.56 ± 1.50	16.39 ± 1.61	16.36 ± 1.59	16.94 ± 1.22	4.061	0.018
Sex, *n* (male/female)	117/139	40/44	39/49	38/46	0.200	0.905
Height, cm	165.18 ± 7.31	164.41 ± 7.43	165.04 ± 7.25	166.09 ± 7.23	1.144	0.320
Weight, kg	63.46 ± 14.19	62.39 ± 13.20	61.97 ± 15.08	66.10 ± 13.98	2.207	0.112
BMI, kg/m^2^	23.16 ± 4.37	23.01 ± 4.24	22.62 ± 4.53	23.86 ± 4.28	1.819	0.164

Values are presented as mean ± SD. BMI, body mass index; FHP, forward head posture.

**Table 2:** Differences in postural variables across FHP tertiles.

Characteristic	Total (*n* = 256)	Lower FHP Tertile (*n* = 84)	Middle FHP Tertile (*n* = 88)	Upper FHP Tertile (*n* = 84)	Partial η^2^	*p*
Sagittal plane						
Head posture ^a,b,c^, cm	4.83 ± 1.78	2.89 ± 0.85	4.76 ± 0.41	6.84 ± 0.98	0.808	<0.001
Rt. Protracted shoulder ^d^, cm	2.99 ± 2.55	2.38 ± 2.74	3.26 ± 2.23	3.32 ± 2.57	0.029	0.023
Lt. Protracted shoulder ^d^, cm	3.25 ± 2.67	2.57 ± 2.88	3.42 ± 2.29	3.76 ± 2.72	0.028	0.030
Trunk lean ^a,c,e^, degree	−4.43 ± 1.72	−5.33 ± 1.85	−4.43 ± 1.33	−3.53 ± 1.72	0.170	<0.001
Frontal plane						
Rt. Eye level, cm	147.11 ± 7.46	146.07 ± 7.62	147.52 ± 7.43	147.73 ± 7.29	0.007	0.395
Lt. Eye level, cm	146.71 ± 7.42	145.90 ± 7.56	146.98 ± 7.50	147.24 ± 7.21	0.004	0.586
Rt. Shoulder level, cm	124.89 ± 6.02	123.65 ± 6.02	125.49 ± 5.92	125.50 ± 6.00	0.018	0.097
Lt. Shoulder level, cm	124.56 ± 6.11	123.40 ± 6.07	124.88 ± 6.07	125.38 ± 6.08	0.014	0.169
Trunk side bending, degree	0.58 ± 2.18	0.45 ± 2.20	1.06 ± 2.07	0.21 ± 2.20	0.020	0.078
Rt. Pelvic level, cm	76.19 ± 4.41	75.30 ± 4.40	76.72 ± 4.16	76.53 ± 4.59	0.021	0.084
Lt. Pelvic level, cm	75.82 ± 4.43	74.87 ± 4.55	76.34 ± 4.18	76.23 ± 4.47	0.020	0.066
Transverse Plane						
Head Rotation	−2.04 ± 9.18	−1.49 ± 8.52	−2.00 ± 8.60	−2.64 ± 10.40	0.001	0.918
Trunk Rotation	−0.23 ± 3.86	−0.23 ± 3.75	0.62 ± 3.79	−1.13 ± 3.88	0.019	0.093
Pelvic Rotation	1.27 ± 4.74	1.20 ± 4.61	1.72 ± 5.07	0.87 ± 4.53	0.004	0.595

Values are presented as mean ± SD. Negative trunk lean values indicate posterior inclination; values closer to zero indicate less posterior inclination. Because right- and left-side trunk lean values were identical, trunk lean is presented as a single variable. *p* values and partial η^2^ are from follow-up univariate ANCOVAs after a significant age-adjusted MANCOVA; pairwise comparisons were Bonferroni-adjusted. ^a^ Lower vs. Middle (*p* < 0.001); ^b^ Middle vs. Upper (*p* < 0.001); ^c^ Lower vs. Upper (*p* < 0.001); ^d^ Lower vs. Upper (*p* < 0.05); ^e^ Middle vs. Upper (*p* < 0.01).

**Table 3:** Univariate and age-adjusted multivariable linear regression analyses for forward head posture.

Variable	Univariate Analysis B (95% CI)	β	*p*	Adjusted Multivariable Analysis B (BCa 95% CI) *	β	*p* *	VIF
Age	0.134 (−0.012–0.280)	0.113	0.072	0.062 (−0.051–0.169)	0.052	0.277	1.131
Rt. Protracted shoulder	0.053 (−0.033–0.139)	0.076	0.226	–	–	–	
Lt. Protracted shoulder	0.063 (−0.021–0.148)	0.095	0.142	–	–	–	
Trunk lean,	0.416 (0.298–0.535)	0.401	<0.001	0.408 (0.279–0.526)	0.393	0.001	1.049
Rt. Eye level	0.018 (−0.012–0.048)	0.076	0.241	–	–	–	
Lt. Eye level	0.012 (−0.019–0.043)	0.052	0.430	–	–	–	
Rt. Shoulder level	0.036 (−0.001–0.073)	0.121	0.057	–	–	–	
Lt. Shoulder level	0.038 (0.002–0.075)	0.131	0.041	−0.012 (−0.075– 0.056)	−0.015	0.805	3.890
Trunk side bending	−0.042 (−0.146–0.061)	−0.052	0.420	–	–	–	
Rt. Pelvic level	0.054 (0.005–0.104)	0.135	0.031				
Lt. Pelvic level	0.055 (0.005–0.104)	0.136	0.030	0.039 (−0.013–0.091)	0.098	0.136	3.652
Head Rotation	0.003 (−0.021–0.028)	0.018	0.784	–	–	–	
Trunk Rotation	−0.030 (−0.093–0.034)	−0.064	0.358	–	–	–	
Pelvic Rotation	−0.024 (−0.071–0.022)	−0.064	0.303	–	–	–	

* Multivariable regression coefficients are original-sample estimates; corresponding *p* values and BCa 95% confidence intervals were obtained from 1000 participant-level bootstrap samples. Rt. pelvic level was excluded from the final multivariable model because of severe multicollinearity with Lt. pelvic level when both variables were entered simultaneously (VIF = 23.176 and 22.044, respectively). Sensitivity analyses including Rt. pelvic level separately showed similar results.

**Table 4:** Sensitivity analysis additionally adjusted for sex, height, and BMI.

Variable	B 95% CI *	*p* *
Age	0.038 (−0.099–0.175)	0.571
Sex	0.310 (−0.242–0.840)	0.264
Height	0.014 (−0.039–0.077)	0.640
BMI	0.015 (−0.036–0.067)	0.543
Trunk lean	0.412 (0.284–0.534)	0.001
Lt. shoulder level	−0.011 (−0.116–0.088)	0.829
Lt. pelvic level	0.048 (−0.055–0.150)	0.355

* Multivariable regression coefficients are original-sample estimates; corresponding *p* values and BCa 95% confidence intervals were obtained from 1000 participant-level bootstrap samples.

## Data Availability

The datasets generated and analyzed during the current study are available from the corresponding author upon reasonable request.
